# Strategies for editors to contribute for the achievement of the Sustainable Development Goals by 2030

**DOI:** 10.1590/1518-8345.0000.4059

**Published:** 2023-09-25

**Authors:** Lilian Nassi-Calò

**Affiliations:** 1 Consultora del Programa SciELO.


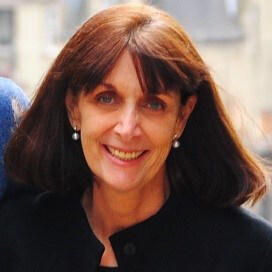


The 2030 Agenda for Sustainable Development adopted by all Member States of the United Nations in 2015 potentially represents peace and prosperity for people and the planet, now and in the future. Its 17 Sustainable Development Goals (SDGs), which include 169 targets, are broad and ambitious, as well as interdependent, covering issues of social and economic development, gender equality, water and sanitation, energy, urbanization, the environment, and social justice, among others^(^(1)^)^.

In September 2023, world leaders will meet in New York to try to find an emergency plan, since forecasts indicate that halfway through the deadline for achieving them, none of the goals and only 12% of the targets will be reached^(^(2)^)^. The failure to achieve the SDG targets is not for lack of trying. Researchers around the world have aligned their work with the SDGs, along with global efforts such as UN conventions on climate change and biodiversity loss. Unfortunately, fractured global geopolitics have limited international cooperation, as well as coordination across topics and disciplines.

Furthermore, there are potentially conflicting relationships between the SDGs, as actions that could favor one of the goals sometimes have negative effects on others. For example, actions to develop alternative energy sources (SDG 7) to reduce climate change (SDG 13) can have a negative effect on local biodiversity (SDGs 14 and 15) through the construction and operation of solar and wind power plants. As much as financing fossil sources for energy generation can create jobs and promote economic growth (SDG 8), these sources have a negative impact on health and well-being (SDG 3).

Every four years, the United Nations (UN) commissions a Global Sustainable Development Report from independent scientific advisors, which serves to point out where the SDGs are failing and what can be done to rescue them. These documents reiterate the need for transformational change to put the planet back on a sustainable path.

The 2019 Report organized the implementation of the 17 SDGs into six “transformations” and established basic premises for achieving them. The six axes are (1) Education, gender and inequalities; (2) Health, well-being and demographics; (3) Decarbonization of energy and sustainable industry; (4) Sustainable food, water, land and oceans; (5) Sustainable cities and communities; and (6) Digital transformation and sustainable development^(^(3)^)^. Each transformation describes a major change in the organization of social, political, and economic activities that transforms the use of resources, the role of institutions, technologies, and social relations to achieve the main outcomes of the SDGs. “*The (2019) report clearly shows that such transformations are possible, and that sufficient knowledge is available to get started. However, we need to overcome the gap between what we know and what is being done. We strongly believe that scientific evidence must contribute to triggering the social and political debates about the hard choices that need to be made, and to formulating effective policies for the necessary transformations.*”^(^(4)^)^

The 2023 report (in preparation) points out that the path to sustainability must necessarily include the elimination of unsustainable practices, even considering the resulting social and economic cost. For example, developing clean energy sources is not enough if fossil fuels are not phased out. The necessary transformations require huge financial resources, requiring public and private investments of around US$ 2.5 trillion by 2030. However, not only financial resources are needed for the efforts to be successful. New forms of governance must be adopted, with the creation of new institutions and the reform of old ones, prioritizing sustainability. Moreover, the people involved must be equipped with the right resources and skills to bring about change, and this will be especially important in low- and middle-income countries.

Efforts to achieve the goals of the SDGs have already brought about worldwide transformations in the way science is done, which is becoming increasingly equitable and inclusive, openly shared, reliable, and socially robust, in other words, responsive to social context and needs. Knowledge needs to be increasingly accessible, not only to the scientific community, but also to society. An independent UN study published in 2021 shows that science in low- and middle-income countries is already much more aligned with the SDGs and that these countries have published a much higher volume of research related to the SDGs than high-income countries^(^(5)^)^.

Scientific publishing plays a crucial role in achieving the SDGs, facilitating the dissemination of research results, promoting evidence-based practices, and fostering collaborations between institutions and countries. In particular, SDGs related to health and well-being can benefit from:

1. Broad sharing of research results and knowledge. The dissemination of research results to society as a whole plays a vital role in advancing the understanding of health issues, promoting evidence-based practices, and informing the formulation of public policies.
2. Improving access to information through open access publishing facilitates the implementation of evidence-based interventions and promotes self-care.
3. Tackling global health challenges. By prioritizing the publication of research that addresses health challenges such as infectious diseases, maternal and child health, non-communicable diseases and environmental health, scientific editors contribute directly to achieving the health-related SDGs.
4. Encouraging interdisciplinary collaboration between researchers can lead to innovative solutions to complex health issues and promote comprehensive approaches to achieving the health-related SDGs.
5. Monitoring progress and evaluating interventions. Scientific journals allows researchers to publish studies that evaluate the effectiveness of interventions, analyze progress towards specific health goals and identify gaps in the approaches adopted. This knowledge makes it possible to adapt strategies and allocate resources effectively.
6. Dissemination of good practices. Scientific journals publish research on good practices, successful interventions and lessons learned in different regions and contexts, which can be replicated in other regions, accelerating progress towards the health-related SDGs.
7. Stakeholder and community engagement. The publications encourage dialog involving stakeholders, policymakers, and health professionals through comments, (open) peer reviews and messaging, allowing different voices to contribute to the discourse and ensuring that interventions are contextually appropriate and meet the needs of the communities they are intended for.
8. Adopting open science practices such as publishing in open access, posting results as preprints, making underlying research data available in open access repositories, and carrying out open peer review broadens the discovery of research results to society as a whole and not just to researchers and health professionals, while at the same time increasing research reliability and reproducibility.
9. In general, scientific publishing plays a vital role in promoting knowledge exchange, evidence-based decision-making and collaboration, crucial premises for achieving the health-related SDGs.

If the world is to accelerate the achievement of the SDGs by 2030, society as a whole must be aware of its role. It is in our hands as citizens, researchers, science editors, professors, and health professionals to contribute to achieving most of them.
